# Cellular pharmacokinetic and pharmacodynamic analyses of ethacrynic acid: Implications in topical drug delivery in the eye

**Published:** 2011-09-27

**Authors:** Cheng-Wen Lin, Pedro Gonzalez, Fan Yuan

**Affiliations:** 1 Department of Biomedical Engineering, Duke University, Durham, NC; 2 Department of Ophthalmology, Duke University, Durham, NC

## Abstract

**Purpose:**

Ethacrynic acid (ECA) is a potential trabecular meshwork (TM) drug that has shown promising results in preclinical studies for treatment of primary open-angle glaucoma. However, topical application of ECA is currently limited by adverse effects in corneal tissues. To this end, we developed a new theoretical model to evaluate time-dependent toxicity induced by ECA in corneal epithelial cells.

**Methods:**

The model consisted of a cellular pharmacokinetic (PK) module to determine intracellular concentration of ECA, and a pharmacodynamic (PD) module to determine the cytotoxicity of ECA. It was assumed that ECA-induced cytotoxicity depended on drug exposure time and peak concentration of bound ECA in cells. In addition to the model development, we experimentally determined the intracellular concentration of ECA as a function of drug dose and treatment time.

**Results:**

The intracellular concentration increased linearly (i.e., no saturation) with increasing the dose of ECA. It also increased initially with time and then reached a steady-state at ~40 min. The percent of cells survived after treatment decreased with increasing the dose of drug or the time of treatment. The experimental data were fit by the new PK and PD models to obtain values of model constants. One of the unique applications of these models was to predict cell survival relative to control when extracellular concentration of ECA varied with time. The prediction showed that the toxicity of ECA might be significantly overestimated by using the traditional LC_50_ determined in vitro.

**Conclusions:**

The new PK and PD models developed in this study were capable to fit experimental data and predict time-dependent toxicity of ECA in corneal epithelial cells. The models may be useful for optimizing the dose and schedule in topical application of ECA for glaucoma treatment.

## Introduction

Ethacrynic acid (ECA), a potential trabecular meshwork (TM) drug, has shown promising results in pre-clinical studies to treat primary open-angle glaucoma [1-6]. The efficacy of treatment depends on how much ECA can be delivered to TM tissues. Although different approaches to drug delivery to the anterior chamber have been developed [7-10], the preferred choice is still the topical application because of its non-invasiveness and convenience in the clinic. The efficiency of topical application is currently limited by adverse effects of drugs in corneal tissues observed at the dose required for achieving a therapeutic concentration in the TM [6,11]. To overcome the toxicity problem, it is important to understand mechanisms of toxicity in corneal epithelial cells and develop novel techniques to accurately evaluate the toxicity. A widely used parameter for toxicity evaluation in vitro is the lethal concentration at which 50% of cells are killed (LC_50_) when the cells are continuously exposed to the drug for a certain period. If extracellular concentration of a drug varies significantly with time, which often happens in vivo, the LC_50_ becomes meaningless. In this case, other quantities need to be considered for the evaluation of drug toxicity. For example, one can quantify the toxicity by using the area-under-the-curve (AUC) at which 50% of the cells are killed after treatment (AUC_50_). Experimentally, it is feasible to determine LC_50_ or AUC_50_ by treating the cells of interest with specific drugs for a short period (e.g., a few hours), but it is difficult to perform long-term (e.g., a few weeks) experiments. This is because primary cells have only limited life span in culture and immortalization of these cells may cause changes in their characteristics. One alternative approach to addressing the long-term toxicity issue is to develop cellular pharmacokinetic (PK) and pharmacodynamic (PD) models and used them to simulate dose response curves in terms of cell survival under different experimental conditions.

The development of PK models can be straightforward since drug transport and reactions are governed by general principles. On the other hand, PD models depend on mechanisms of drug actions in cells and molecular properties of drugs, which may be unknown in many cases. Despite of this challenge, various PD models have been developed to predict how cell survival relative to the control, S, depends on drug concentration and treatment period. Quantitatively, S is defined as the number of cells survived after drug treatment divided by the number of live cells in untreated control. The drug concentration in a PD model may refer to intracellular concentration, extracellular concentration, or the combination of the two. When the concentration is time-dependent, it may refer to peak concentration. Furthermore, S is an explicit function of drug concentration and exposure time in some models but an implicit function in other models where concentration and time are included through AUC or other quantities (see the Methods section) [12-15]. In many studies, S is assumed to be a sigmoidal function that can be approximated by a Hill-type Equation [13,14].

The goal of this study was to develop a new theoretical framework consisting of cellular PK and PD modules. The new model can be used to investigate ECA induced toxicity in corneal tissues, and to facilitate development of novel strategies for improving topical drug delivery by combining it with mathematical models of ECA transport in the eye [16].

## Methods

### Cell culture

Corneal epithelium was removed carefully from corneal grafts in fresh enucleated porcine eyes, using a surgical scalpel, and placed into a 50 ml beaker with Hank’s balanced salt solution (HBSS; Gibco, Grand Island, NY) and 2% penicillin/streptomycin at room temperature. After 5 min, the corneal epithelium was digested using protease (Sigma, St. Louis, MO) for 60 min, and the solution was centrifuged at 704× g for 5 min to remove the enzyme. The corneal epithelium was further digested using collagenase (Type IA; Sigma) for 90 min. After digestion, the solution was filtered through a sterile cell strainer (40 μm Nylon; BD Biosciences, Franklin Lakes, NJ) and centrifuged at 704× g for 5 min. The primary porcine corneal epithelial cells (PCEC) harvested were then re-suspended in culture medium containing DMEM-F12 (BioWhittaker, Walkersville, MD), 10% fetal bovine serum (FBS), 0.02 mg/ml gentamicin, 2 mM L-glutamine, 0.5% dimethyl sulfoxide (DMSO), 5 μg/ml insulin, 1% penicillin and streptomycin, and 10 ng/ml recombinant human epidermal growth factor (EGF), and cultured at 37 °C with 5% CO_2_ and 95% humidified air. After reaching confluence in the flask, the cells were transferred onto 24-well plates at a density of 10^5^ cells per well, and cultured for three to five days until confluent monolayers were formed. At this stage, the cell density was approximately 4×10^5^ cells per well. These cells at the first passage still retained the epithelial phenotype. Thus they were used in all experiments described below.

### Uptake of ECA by primary corneal epithelial cells

Confluent primary PCECs were cultured in 24-well plates. Before adding ECA solutions, the culture medium was removed, and the cells were washed three times with HBSS to remove the remaining culture medium. The cells were then exposed to 1 ml HBSS containing different concentrations of ECA (50, 100, 200, 300, and 600 μM) for varying periods (0.5, 2, 8, 15, and 45 min). After ECA treatment, the drug solution in each 24-well plate was collected for offline analysis. The cells were washed twice with fresh HBSS; and the resulting solutions were also collected for offline analysis. All experiments were performed in triplicate.

The three solution samples collected in the experiment described above were analyzed by high-performance liquid chromatography (HPLC). The data showed that the amount of ECA in the third sample was negligible, and that there was a significant ECA loss in the second sample, compared with ECA concentration in the first one. The question was, how much of the ECA loss was due to ECA internalization by cells? In a previous study, Tirona et al. [17] observed that when hepatocytes were treated with ECA in vitro, the loss of extracellular ^14^C-ECA was completely accounted for by the formation of ECA-glutathione (GSH) conjugate within cells, and that the total mass of ECA was conserved throughout the entire treatment period. Therefore, we assumed that the amount of cellular uptake of ECA was equal to the amount of loss of extracellular ECA. Based on this assumption, the reported intracellular concentration was calculated as the total amount of extracellular ECA loss divided by the total volume of cells in the monolayer.

### Determination of ECA concentration by HPLC

**Figure 1:**
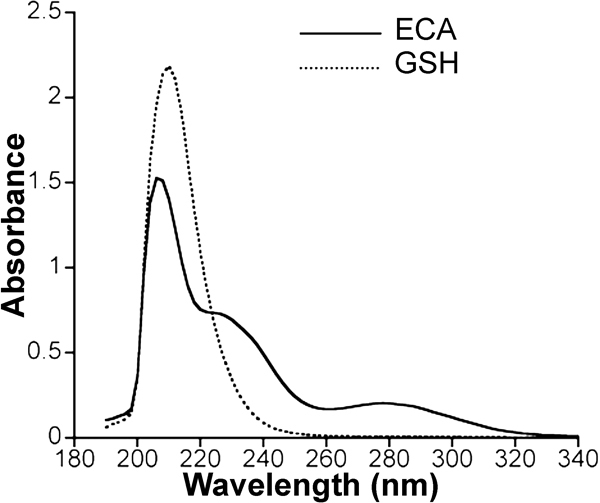
UV absorbance spectrums of ECA and GSH. The concentrations of ECA and GSH were 50 μM and 600 μM, respectively. The spectrum curves suggested that ECA concentration should be measured at 270 nm to minimize possible interference with signals from GSH.

The HPLC measurement was performed using an Inertsil ODS-3 5U, 4.6 mm i.d.×250 mm (Varian Inc., Palo Alto, CA) with a mobile phase, H_2_O: acetonitrile: acetic acid (49:49:2 by volume), at flow rate of 1.0 ml/min. The detector for UV absorbance was set at 270 nm instead of 230 nm due to possible interference of ECA spectrum with that of GSH at 230 nm (see Figure 1). All sample solutions were filtered through a 0.2 μm AcroPrep^TM^ 96 filter plate (Pall Corporation, Ann Arbor, MI) before being transferred into a 384-well plate for HPLC analysis. The volume of each injection was 50 μl, and the running time was 30 min. System calibration was performed for each individual HPLC analysis by comparing the peaks of absorbance to the known concentrations of ECA. All measurements were performed within the linear ranges of the calibration curves. The concentration in each sample was first determined in μM and then converted to nmol/(10^5^ cells) in 24-well plates, based on sample volume and cell density in each well.

### Determine cytotoxicity of ECA in porcine corneal epithelial cells in vitro

Primary PCECs at the first passage were placed in 96-well plates at a density of 1×10^4^ cells/well. After 24 h, the culture medium was replaced with 100 μl of fresh medium containing ECA at different concentrations: 0, 10, 30, 50, 75, 100, 200, 300, or 600 μM. The cells were treated for 2, 6, 24, or 72 h. After treatment, cell viability in all groups was measured by using CytoTox 96 kit (Promega, Madison, WI). The ratio of the numbers of viable cells between treated and untreated (i.e., at zero concentration of ECA) groups is reported as the survival relative to control (S). All assays were performed in quadruplicates and the mean value is reported in this paper.

### Cellular PK model

**Figure 2:**
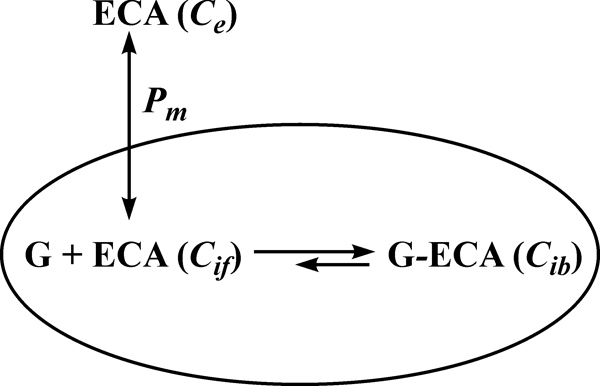
Schematic representation of the proposed pharmacokinetic model. The definition of each term is as follows: *C_e_*_:_ extracellular ECA concentration; *C_if_*: intracellular concentration of free (unbound) ECA; *G*: intracellular binding site available to ECA; *G-ECA*: bound ECA; *C_ib_*: intracellular concentration of bound ECA; and *P_m_*: permeability coefficient of cell membrane.

A cellular PK model was proposed to determine the intracellular and extracellular concentrations of ECA under different conditions. The schematic representation of the model is shown in Figure 2. The model development was based on the following considerations. First, ECA can be metabolized in cells [18,19]. Yamamoto et al. [19] have measured concentrations of ECA metabolites in isolated rat hepatocytes. The data demonstrate that they are significantly lower than ECA concentration after cells are treated with the drug for 30 min, and the metabolic rate can be reduced by an inhibitor of cytochrome P450. In this study, we used corneal epithelial cells, which were expected to metabolize ECA at a lower rate, compared to hepatocytes. Thus, ECA metabolism was neglected in our PK model. Second, ECA in solutions can be degraded as well. However, data in the literature showed that ECA at 0.1 mg/ml was stable in 100 mM potassium phosphate buffer at pH 7.4 for at least 6 h [20]. In another study, only 17% of ECA at 0.05 mg/ml was degraded after the solution was stored for 480 h at 60 °C and pH 8 [21]. Significant degradation of ECA occurred only at pH 10 and pH 12. In our experiments, we only used fresh ECA solutions prepared on the same day, and all samples were analyzed by HPLC immediately after cell incubation. Thus, extracellular degradation of ECA was negligible. Third, different mechanisms can be involved for cellular uptake of ECA. Active transport may exist in liver cells [22], but is insignificant in confluent rat renal proximal tubular cells [23], and cells in rabbit kidney cortex [24]. Active transport can be modeled in pharmacokinetic analysis by adding a saturable component in the equation for rate of cellular uptake [17]. However, this addition would be inconsistent with our experimental data obtained in corneal epithelial cells (see the Results section), which showed that the uptake rate was approximately a linear function of extracellular concentration of ECA. Therefore, the saturable component or active transport was neglected in our PK model. Fourth, efflux of ECA-glutathione conjugate has been considered in PK analysis of ECA using rat hepatocytes [17]. It was an order of magnitude slower than the passive diffusion of ECA. Thus, efflux of ECA conjugated with endogenous molecules (e.g., GSH) was neglected in our PK model as well. Taken together, we assumed that ECA entered into and moved out of cells through passive diffusion. As a result, the mass balance for ECA in cells is governed by Equation 1;

$$\frac{dC_{it}}{dt}=P_{m}(C_{e}-C_{if})\text{ (1)}$$

where *C_it_* is the total intracellular concentration of ECA; *C_e_* is the extracellular ECA concentration; *C_if_* is the intracellular concentration of free (unbound) ECA; and *P_m_* is the permeability coefficient of cell membrane. Within the cell, ECA could bind to various intracellular targets (e.g., GSH), which were lumped together and represented by G in the PK model (see Figure 2). Previous studies have shown that binding of ECA to GSH is significantly faster than its diffusion into cells [17,20]. Therefore, binding was assumed to be at a quasi-equilibrium state, and the equilibrium concentration of bound ECA (*C_ib_*) was determined by Equation 2;

$$C_{ib}=\frac{B_{\max}C_{if}}{K_{D}+C_{if}}\text{ (2)}$$

where *B_max_* is the maximum concentration of intracellular binding sites for ECA and *K_D_* is the equilibrium dissociation constant. The total intracellular ECA concentration was the sum of *C_if_*  and *C_ib_*;

$$C_{it}=C_{if}+C_{ib}\text{ (3)}$$

Substituting Equations 2 and 3 into Equation 1 yields;

$$\frac{dC_{if}}{dt}=P_{m}\left[\frac{{\left(K_{D}+C_{if}\right)}^{2}}{{\left(K_{D}+C_{if}\right)}^{2}+K_{D}B_{\max}}\right]\left(C_{e}-C_{if}\right)\text{ (4)}$$

For a given dose or initial concentration of ECA in the extracellular medium (C_e0_), C_e_ was determined by using the mass balance equation;

$$C_{e}V_{o}+(C_{if}+C_{ib})V_{i}=C_{eo}V_{o}\text{ (5)}$$

where *V*_o_ is the volume of extracellular ECA solution, *V_i_* is the total volume of intracellular space, which was calculated based on the number of cells per well, assuming that the cell was spherical and had a diameter of 21 μm [25]. Substituting Equations 3 and 5 into Equation 1 yields;

$$\frac{dC_{if}}{dt}=\frac{P_{m}}{V_{0}}\left[\frac{{\left(K_{D}+C_{if}\right)}^{2}}{{\left(K_{D}+C_{if}\right)}^{2}+K_{D}B_{\max}}\right]\;\left[C_{eo}V_{o}-\left(C_{if}+\frac{B_{\max}C_{if}}{K_{D}+C_{if}}\right)V_{i}-C_{if}V_{o}\right]\text{ (6)}$$

Equation 6 was solved numerically to obtain *C_if_*, which was then substituted into Equations 2 and 3 to obtain *C_it_*. The values of *P_m_*, *K_D_*, and *B_max_* were determined by fitting the experimental data with the predicted values of *C_it_* using a nonlinear least square method (MATLAB software; The Mathworks Inc., Natick, MA).

### Cellular PD model

To evaluate survival of primary PCECs treated with ECA under different conditions, a cellular PD model was developed based on the Hill-type equation;

$$S=\frac{1}{1+Ax^{m}}\text{ (7)}$$

where *A* and *m* are constants. The quantity *x* is a function of drug concentration and exposure time, which was determined based on the following considerations. It has been proposed that ECA-induced cytotoxicity is mediated partly through depletion of GSH in both cytosol and mitochondria, which has been observed experimentally in hepatocytes [19,26], cerebellar astrocytes [27], and motor neurons [28,29]. When the GSH level is reduced to 20%–30% of its normal level, a large amount of reactive oxygen species are produced in cells, which may cause cell death [29]. It has been hypothesized that ECA can deplete intracellular GSH through three mechanisms: (1) direct (non-enzymatic) interaction with GSH to form thiol adducts through a Michael-type reaction; (2) conjugation with GSH catalyzed by glutathione S-transferases (GSTs) [19,27,30]; and (3) inhibition of GSH biosynthesis through irreversible conjugation with glutathione reductase, which is an enzyme that catalyzes the reduction reaction to convert glutathione disulfide (GSSG) to GSH [19,30-32]. Experimental data in the literature have also suggested that other intracellular protein sulfhydryls may contribute to the ECA-induced cytotoxicity. For instance, microtubule and F-actin assembly in cells can be affected by ECA [33-38]. Motor neurons exhibit chromatin condensation and nuclear fragmentation at 3 h post ECA treatment at 100 µM [28], indicating that DNA is also an intracellular alkylating targets of ECA.

Based on the discussion above, *x* in Equation 7 was assumed to be a function of the peak concentration of bound ECA in cells, (C_ib_)_p_, and the time, t_p_, at which C_ib_ reached its peak level, i.e., x=f[t_p_, (C_ib_)_p_]. If C_ib_ was increased gradually and reach a plateau, there would be no peak concentration. In this case, (C_ib_)_p_ would refer to the maximum value of C_ib_, and t_p_ would be the total treatment period. Various forms of f have been proposed in previous studies for fitting experimental data [13]. The form choice is not necessarily to be unique for a given set of data. In this study, we assumed that f was a power function of concentration and time;

$$x=\frac{{\left(C_{ib}\right)}_{p}}{B_{\max}}t_{p}^{n}\text{ (8)}$$

where *n* is a constant and the power raised for (C_ib_)_p_ can be absorbed into *m* in Equation 7. Thus, it is not explicitly shown in Equation 8. Substituting Equation 8 into Equation 7 yielded a new equation that was used to fit the cytotoxicity data obtained in this study to determine the values of three constants, *A*, *n*, and *m*, using the nonlinear least square method in MATLAB software (The Mathworks Inc.). Similar to most PD models in the literature, our model assumed implicitly that the values of *P_m_*, *K_D_*, and *B_max_* determined in a relatively short-term PK experiment were still valid in the cytotoxicity analysis.

### Simulation of drug toxicity due to transient exposure to ECA

The PK and PD models would be applied to predicting ECA toxicity in corneal tissues in the following two scenarios of drug delivery: (i) topical application of ECA as eye drops and (ii) sustained release of ECA in the pre-corneal region from a copolymer film containing 21% Pluronic^®^ F127 and 10% Pluronic^®^ F68 [39]. In each scenario, drug clearance in the pre-corneal region was approximated by the first-order kinetics. The estimated half-lives for these scenarios were 3.0 min and 30.3 min, respectively [39,40]. The concentrations of ECA in the pre-corneal region in the first and second scenarios were given by;

$$C_{e1}=C_{e0}e^{-0.231t}\text{ for t>0 (9)}$$

$$C_{e2}=C_{e0}e^{-0.0229t}\text{ for t>0 (10)}$$

respectively, where C_e0_ is the initial ECA concentration and *t* is the exposure time.

## Results

**Figure 3:**
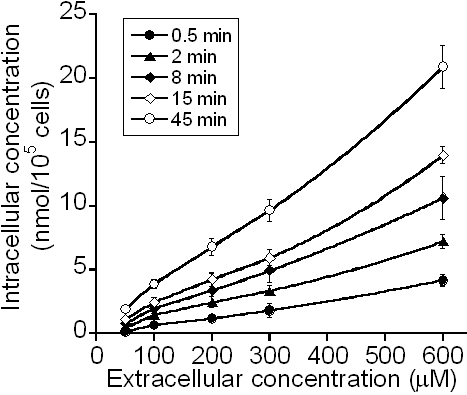
Intracellular concentration of ECA. It was a function of the initial extracellular concentration of ECA and the period of treatment.

Figure 3 shows the intracellular concentration of ECA (in nmol/10^5^ cells; C_it_) after PCECs were treated with ECA for different time periods. It is plotted as a function of the initial concentration of ECA in extracellular medium (C_e0_). The data demonstrated that C_it_ was considerably higher than the corresponding value of C_e0_, which in turn was higher than the extracellular concentration of ECA at the end of treatment, suggesting that the majority of ECA molecules in cells were bound to intracellular substrates. The data also showed that the intracellular concentration was linearly dependent on C_e0_ (i.e., no saturation) if the drug exposure time was fixed, suggesting a very high concentration of binding sites available to ECA in cells.

**Figure 4:**
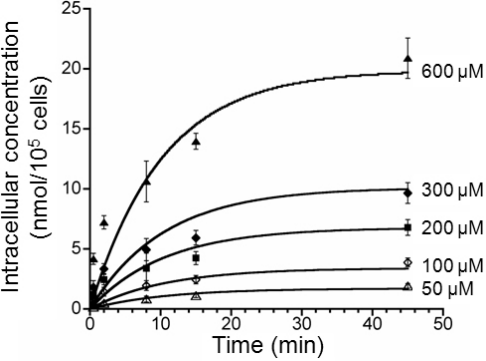
Time dependent changes in intracellular concentration of ECA. The symbols indicate experimental data and the curves were resulted from fitting the PK model to the data. The best-fit values of the model constants are reported in Table 1.

The data shown in Figure 3 were re-plotted to show time-dependent changes in intracellular concentration of ECA (see in Figure 4). The data were fit by the PK model described in the Methods section; and the best-fit values of the model constants are reported in Table 1. The concentration profiles indicated that the rate of cellular uptake of ECA, i.e., the slopes of the curves, decreased with increasing the drug exposure time, which was expected since the diffusion of ECA into cells would reduce concentration difference across the cell membrane. The concentrations reached steady-states at ~40 min.

**Table 1:** Values of fitted PK and PD model constants.

**Constant**	**Description**	**Value**
P*_m_*	Permeability coefficient of membrane	0.003213 ml/(min 10^5^ cells)
K*_D_*	Equilibrium constant for GSH conjugation	13,800 μM
B*_max_*	Maximum substrate concentration	541 nmol/10^5^ cells
*A*	Constant in the PD model	1.53×10^13^
*n*	Constant in the PD model	0.3^a^
*m*	Constant in the PD model	7.48^a^

^a^The values of *m* and *n* depended on the choice of time unit. It was required to be “hour” for the values listed here.

**Figure 5:**
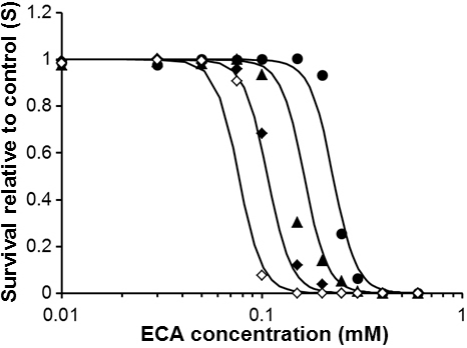
Experimental data of cell survival relative to control (S). It was a function of the initial extracellular concentration of ECA and the period of treatment. The symbols indicate experimental data and the curves were resulted from fitting the PD model to the data. ●: 2 h of constant ECA exposure; ▲: 6 h of constant ECA exposure; ◆: 24 h of constant ECA exposure; ◇: 72 h of ECA exposure. The best-fit values of the model constants are reported in Table 1.

The number of PCECs survived after ECA treatment was quantified and normalized by the number of viable cells in the untreated group. It was a function of the initial concentration of ECA in the extracellular medium and the drug exposure time. Data shown in Figure 4 indicated that *C_if_* and *C_e_* could be assumed to be equal to each other for time longer than 2 h. By using this information and simultaneously solving Equations 2, 5, and 7 described in the Methods section, S could be expressed as a function of *C_e0_* and *t_p_*. This expression was then used to fit the experimental data obtained in this study, which yielded the values of constants in the PD model: A=1.53×10^13^, n=0.3, and m=7.48 if the unit of time was hour. The results of curve-fitting are plotted in Figure 5. The PD model predicted that the LC_50_ of ECA, in terms of C_e0_, were 227, 163, 107, and 77 µM for the PCECs treated continuously with ECA for 2, 6, 24, and 72 h, respectively.

**Figure 6:**
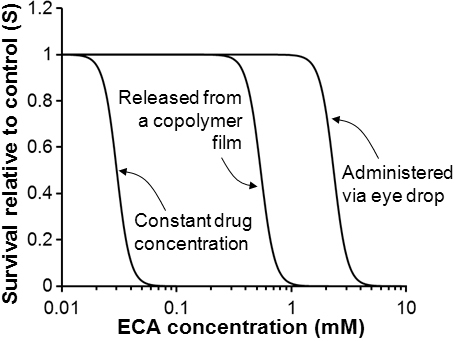
Cell survival relative to control simulated by using the cellular PK and PD models. S was plotted as a function of the initial concentration of ECA in the extracellular medium for three different scenarios of topical drug delivery in the eye. (i) ECA was applied via traditional eye drops (LC_50_=2330 μM); (ii) ECA was released from a Pluronic*^®^* copolymer film (LC_50_=540 μM); and (iii) ECA concentration at corneal surface was maintained at constant levels for 1,000 h (LC_50_=35 μM).

To simulate ECA toxicity in corneal epithelial cells when the drug was administered topically on the corneal surface for glaucoma treatment, Equations 9 and 10 were used to determine C_e_, the concentration of ECA in the pre-corneal region. Two scenarios of topical drug delivery were considered here: traditional eye drop and sustained release of ECA from Pluronic*^®^* copolymer film. The time dependent profiles of C_if_ in epithelial cells on the cornea surface were determined by solving Equation 4, where C_e_ was a given function of time. Substituting C_if_ into Equation 2, the peak level of C_ib_ and the corresponding t_p_ were determined, which were both functions of C_e0_. These values were then substituted into Equation 7 to determine the survival relative to control, S. The simulation results are shown in Figure 6. To demonstrate the difference in cell survival curves between constant and transient exposures of cells to ECA, S was also simulated when the concentration of ECA in the pre-corneal region was fixed at a constant level over a long period (e.g., 1,000 h). It was observed that the survival profile shifted to the left when the exposure time was increased; and the amount of shift diminished if the time was longer than 1,000 h (data not shown). Therefore, we only plotted the survival curve after cells were continuously treated with ECA at a fixed extracellular concentration for 1,000 h. The values of LC_50_ in terms of C_e0_ were calculated; they were 2330 μM, 540 μM, and 35 μM, respectively, for the three different scenarios of drug delivery. When comparing the curves shown in Figure 6 with those in Figure 5, it could be observed that for the same C_e0_, LC_50_ in a transient exposure scenario was several folds higher than the value in the constant exposure scenario, indicating that transient exposure could significantly reduce drug toxicity.

## Discussion

New cellular PK and PD models were developed for investigation of drug toxicity in cells. They could predict the survival relative to control when cells were exposed transiently to the drug. Results from this study suggested that the toxicity of ECA in corneal tissues was significantly overestimated by using the traditional LC_50_ since the clearance of ECA in the pre-corneal region was not considered in previous toxicity studies.

To validate the PK and PD models, we compared results in this study with those in the literature. First, we did the comparison for ECA uptake by different types of cells. Tirona et al. [20] observed that the initial rate of ECA uptake by cultured hepatocytes was 0.62 nmol/(min•10^5^ cells) when the initial concentration of ECA was given at 200 μM in extracellular medium. This data was close to 0.60 nmol/(min•10^5^ cells) observed in this experiment. Haenen et al. [23] investigated ECA uptake by cells after adding 1 ml of ^14^C-labeled ECA solution (14 μM) on top of a monolayer with 2×10^5^ rat renal proximal tubular (RPT) cells. After 30 min, 90% of the radioactivity was recovered from the medium, suggesting that 10% of ECA in the medium was internalized by RPT cells. We did not perform the uptake experiment under the same experimental conditions, but the data could be compared with the PK model prediction. Assuming the initial concentration of ECA, the exposure time, and the cell density to be 14 μM, 30 min, and 2×10^5^ cells per monolayer, respectively, the model predicted that 93% of ECA in the medium above a PCEC monolayer could be recovered, which was close to the data reported in the previous study [23].

Intracellular concentration of unbound ECA was not quantified in this study since it was found that the concentration was below the limit of detection in our system. On the other hand, our PK model predicted that the majority of intracellular ECA molecules were in the form of bound species. The prediction was consistent with those observed in a previous study [17], where the authors demonstrated that the intracellular concentration of unbound ECA was minimal, compared to that of ECA-GSH conjugate.

The data of cellular uptake could further be compared qualitatively to those related to GSH depletion since the latter was caused by the former (see the Methods section). Figure 3 shows that the intracellular concentration of ECA increased approximately linearly with increasing the initial extracellular concentration of ECA for all exposure times, which was consistent with the observation that the loss of GST activity was linearly dependent on the initial extracellular concentration up to 2 mM when cells were treated with ECA for 30 min [41]. Figure 4 shows that the intracellular ECA concentration profiles increased with time initially and then reached a steady-state at ~40 min. If these curves were flipped vertically, they were comparable to the time-dependent profiles of the intracellular GSH concentration in rat hepatocytes treated with ECA [19]. In a similar study, Rizzardini et al. [28] showed that when motor neuron cells were treated continuously with ECA solution at 100 μM, the intracellular GSH level dropped to 25% of its initial level at 1 h and became undetectable within 4 h. Furthermore, Yamamoto et al. [19] demonstrated that intracellular protein sulfhydryls in isolated rat hepatocytes were 30% and 25% of the initial concentration at 1 h and 3 h, respectively, when cells were treated continuously with ECA solution at 1 mM. These data suggested that the rate of GSH depletion was on the same order of magnitude as that of cellular uptake of ECA.

The toxicity data obtained in this study were compared quantitatively with those in the literature. It has been shown that ECA solution at 66 μM had no apparent cytotoxicity for human fibroblasts in vitro when the cells were treated for 2 h. However, the ECA solution became toxic when the treatment was increased to 6 days [42]. To compare with these data, we calculated the LC_50_ values using the PK and PD models. They were 227 μM and 54 μM for PCECs treated with ECA for 2 h and 6 days, respectively, suggesting that the ECA solution at 66 μM was non-toxic to PCECs if the drug exposure time was 2 h but would be severely toxic if the period of treatment was increased to 6 days. In another study, the LC_50_ was determined to be 158 μM when primary bovine TM cells were treated with ECA for 3 h [5]. Using the PK and PD models developed in this study, it was predicted to be 174 μM, which was on the same order of magnitude as that for the TM cells.

The cellular PK and PD models developed in this study can be used to predict ECA toxicity in epithelial cells on the surface of cornea when the drug is applied topically. It has been shown that the ECA concentration in the pre-corneal surface decreases with time after topical application [16]. Thus, the traditional LC_50_ determined with in vitro experiments cannot be used directly to determine drug toxicity in the cornea. To solve this problem, the in vitro data have to be converted to the tissue toxicity using cellular PK and PD models. To illustrate how the new models could be used for this purpose, we simulated the toxicity effect of ECA on epithelial cells when the drug was applied topically on the corneal surface for glaucoma treatment (see Figure 6). The new PK and PD models can also be combined with the mathematical model of ECA transport in the eye, developed in our previous study [16], to improve the prediction on the maximum ECA concentration that can be achieved in the TM (C_TM_). In our previous study, we defined a threshold concentration for toxicity, C_tox_, which was the extracellular concentration of ECA at which a certain percentage (e.g., 2%) of cells would be killed after they were exposed continuously to the drug for 6 h. The value of C_tox_ was found to be approximately 75 μM for corneal epithelial cells [43]. Based on this information, we chose the initial drug concentration (C_e0_) in the pre-corneal surface to be 75 μM. This choice would make sure that the majority of corneal epithelial cells were not killed during the treatment since ECA concentration in the interstitial fluid of cornea could not exceed 75 μM. Under this condition, the ECA transport model would predict that the peak value of C_TM_ was less than 10 μM, which was the minimum effective concentration for improving the outflow facility in human eyes ex vivo [44], no matter whether ECA was released from a Pluronic^®^ copolymer film or applied as eye drops. Therefore, it seemed that ECA was not a good TM drug candidate. However, the conclusion could be different if the new PK and PD models were used to calculate the maximum C_e0_. For example, the new models predicted that the maximum C_e0_ could be increased to 312 μM if ECA was released from the Pluronic^®^ copolymer film. Under this condition, the predicted cell survival relative to control, S, was 98.1% in the cornea, which was the same as the value of S when the cells were treated continuously with an ECA solution at 75 μM for 6 h. Combining the new PK and PD models with the model of ECA transport in the eye developed in the previous study [16], we could predict that although C_TM_ varied with time, it would be higher than the minimum effective concentration (i.e., 10 μM) for approximately 3 h. This analysis demonstrated a unique application of the new cellular PK and PD models for improving ECA delivery to the TM.

### Conclusions

A new theoretical framework was developed in this study that could be used to predict cellular uptake and cell survival relative to control when treated with ECA at different concentrations and for different periods. More importantly, the prediction could be made even when the extracellular concentration varied with time. The model predictions were shown to be consistent with the data reported in the literature. In future studies, the new model can be combined with mathematical models of ECA transport in the eye [16], to simultaneously simulate drug delivery to the TM and long-term toxicity of ECA in corneal tissues. These simulations can be useful for optimizing drug dose and delivery schedule in glaucoma treatment.

## References

[1] Epstein DL, Freddo TF, Bassett-Chu S, Chung M, Karageuzian L (1987). Influence of ethacrynic acid on outflow facility in the monkey and calf eye.. Invest Ophthalmol Vis Sci.

[2] Epstein DL, Hooshmand LB, Epstein MP (1992). Thiol adducts of ethacrynic acid increase outflow facility in enucleated calf eyes.. Curr Eye Res.

[3] Epstein DL, Roberts BC, Skinner LL (1997). Nonsulfhydryl-reactive phenoxyacetic acids increase aqueous humor outflow facility.. Invest Ophthalmol Vis Sci.

[4] Shimazaki A, Ichikawa M, Rao PV, Kirihara T, Konomi K, Epstein DL, Hara H (2004). Effects of the new ethacrynic acid derivative SA9000 on intraocular pressure in cats and monkeys.. Biol Pharm Bull.

[5] Shimazaki A, Suhara H, Ichikawa M, Matsugi T, Konomi K, Takagi Y, Hara H, Rao PV, Epstein DL (2004). New ethacrynic acid derivatives as potent cytoskeletal modulators in trabecular meshwork cells.. Biol Pharm Bull.

[6] Cynkowska G, Cynkowski T, Al-Ghananeem AA, Guo H, Ashton P, Crooks PA (2005). Novel antiglaucoma prodrugs and codrugs of ethacrynic acid.. Bioorg Med Chem Lett.

[7] Wang Y, Challa P, Epstein DL, Yuan F (2004). Controlled release of ethacrynic acid from poly(lactide-co-glycolide) films for glaucoma treatment.. Biomaterials.

[8] Jiang J, Gill HS, Ghate D, McCarey BE, Patel SR, Edelhauser HF, Prausnitz MR (2007). Coated microneedles for drug delivery to the eye.. Invest Ophthalmol Vis Sci.

[9] Kompella UB, Sundaram S, Raghava S, Escobar ER (2006). Luteinizing hormone-releasing hormone agonist and transferrin functionalizations enhance nanoparticle delivery in a novel bovine ex vivo eye model.. Mol Vis.

[10] Prausnitz MR, Noonan JS (1998). Permeability of cornea, sclera, and conjunctiva: a literature analysis for drug delivery to the eye.. J Pharm Sci.

[11] Tingey DP, Ozment RR, Schroeder A, Epstein DL (1992). The effect of intracameral ethacrynic acid on the intraocular pressure of living monkeys.. Am J Ophthalmol.

[12] Kuh HJ, Jang SH, Wientjes MG, Au JL (2000). Computational model of intracellular pharmacokinetics of paclitaxel.. J Pharmacol Exp Ther.

[13] El-Kareh AW, Secomb TW (2003). A mathematical model for cisplatin cellular pharmacodynamics.. Neoplasia.

[14] El-Kareh AW, Secomb TW (2005). Two-mechanism peak concentration model for cellular pharmacodynamics of Doxorubicin.. Neoplasia.

[15] Eichholtz-Wirth H (1980). Dependence of the cytostatic effect of adriamycin on drug concenration and exposure time in vitro.. Br J Cancer.

[16] Lin CW, Yuan F (2010). Numerical simulations of ethacrynic acid transport from precorneal region to trabecular meshwork.. Ann Biomed Eng.

[17] Tirona RG, Tan E, Meier G, Pang KS (1999). Uptake and glutathione conjugation of ethacrynic acid and efflux of the glutathione adduct by periportal and perivenous rat hepatocytes.. J Pharmacol Exp Ther.

[18] Adams DJ, Balkwill FR, Griffin DB, Hayes JD, Lewis AD, Wolf CR (1987). Induction and suppression of glutathione transferases by interferon in the mouse.. J Biol Chem.

[19] Yamamoto K, Masubuchi Y, Narimatsu S, Kobayashi S, Horie T (2002). Toxicity of ethacrynic acid in isolated rat hepatocytes.. Toxicol In Vitro.

[20] Tirona RG, Pang KS (1999). Bimolecular glutathione conjugation kinetics of ethacrynic acid in rat liver: in vitro and perfusion studies.. J Pharmacol Exp Ther.

[21] Yarwood RJ, Moore WD, Collett JH (1985). Liquid chromatographic analysis of ethacrynic acid and degradation products in pharmaceutical systems.. J Pharm Sci.

[22] Peterlik M, Gazda H (1980). Sodium-linked transport of ethacrynic acid by rat liver: possible significance for choleretic action.. Biochem Pharmacol.

[23] Haenen HE, Spenkelink A, Teunissen C, Temmink JH, Koeman JH, van Bladeren PJ (1996). Transport and metabolism of glutathione conjugates of menadione and ethacrynic acid in confluent monolayers of rat renal proximal tubular cells.. Toxicology.

[24] Epstein RW (1972). The binding of ethacrynic acid to rabbit kidney cortex.. Biochim Biophys Acta.

[25] De Paiva CS, Pflugfelder SC, Li DQ (2006). Cell size correlates with phenotype and proliferative capacity in human corneal epithelial cells.. Stem Cells.

[26] Ji B, Ito K, Sekine S, Tajima A, Horie T (2004). Ethacrynic-acid-induced glutathione depletion and oxidative stress in normal and Mrp2-deficient rat liver.. Free Radic Biol Med.

[27] Huang J, Philbert MA (1996). Cellular responses of cultured cerebellar astrocytes to ethacrynic acid-induced perturbation of subcellular glutathione homeostasis.. Brain Res.

[28] Rizzardini M, Lupi M, Bernasconi S, Mangolini A, Cantoni L (2003). Mitochondrial dysfunction and death in motor neurons exposed to the glutathione-depleting agent ethacrynic acid.. J Neurol Sci.

[29] Tukov FF, Rimoldi JM, Matthews JC (2004). Characterization of the role of glutathione in repin-induced mitochondrial dysfunction, oxidative stress and dopaminergic neurotoxicity in rat pheochromocytoma (PC12) cells.. Neurotoxicology.

[30] Burg D, Mulder GJ (2002). Glutathione conjugates and their synthetic derivatives as inhibitors of glutathione-dependent enzymes involved in cancer and drug resistance.. Drug Metab Rev.

[31] Sanchez Alcaraz T, Kerkhofs P, Reichert M, Kettmann R, Willems L (2004). Involvement of glutathione as a mechanism of indirect protection against spontaneous ex vivo apoptosis associated with bovine leukemia virus.. J Virol.

[32] Hoffman DW, Wiebkin P, Rybak LP (1995). Inhibition of glutathione-related enzymes and cytotoxicity of ethacrynic acid and cyclosporine.. Biochem Pharmacol.

[33] Rao PV, Shimazaki A, Ichikawa M, Alvarado JA, Epstein DL (2005). Effects of novel ethacrynic acid derivatives on human trabecular meshwork cell shape, actin cytoskeletal organization, and transcellular fluid flow.. Biol Pharm Bull.

[34] Gills JP, Roberts BC, Epstein DL (1998). Microtubule disruption leads to cellular contraction in human trabecular meshwork cells.. Invest Ophthalmol Vis Sci.

[35] Ludueña RF, Roach MC, Epstein DL (1994). Interaction of ethacrynic acid with bovine brain tubulin.. Biochem Pharmacol.

[36] O'Brien ET, Lee RE, Epstein DL (1996). Ethacrynic acid disrupts steady state microtubules in vitro.. Curr Eye Res.

[37] Walker RA, O'Brien ET, Epstein DL, Sheetz MP (1997). n-ethylmaleimide and ethacrynic acid inhibit kinesin binding to microtubules in a motility assay.. Cell Motil Cytoskeleton.

[38] Xu S, Roychowdhury S, Gaskin F, Epstein DL (1992). Ethacrynic acid inhibition of microtubule assembly in vitro.. Arch Biochem Biophys.

[39] Wei G, Xu H, Ding PT, Li SM, Zheng JM (2002). Thermosetting gels with modulated gelation temperature for ophthalmic use: the rheological and gamma scintigraphic studies.. J Control Release.

[40] Scuderi AC, De Lazzari A, Miano F, Zola P (2002). Residence time of netilmicin in tears.. Cornea.

[41] Phillips MF, Mantle TJ (1993). Inactivation of mouse liver glutathione S-transferase YfYf (Pi class) by ethacrynic acid and 5,5′-dithiobis-(2-nitrobenzoic acid).. Biochem J.

[42] Soltys BJ, Gupta RS (1994). Changes in mitochondrial shape and distribution induced by ethacrynic acid and the transient formation of a mitochondrial reticulum.. J Cell Physiol.

[43] Lin CW. Drug Delivery to the Eye for Treatment of Primary Open-Angle Glaucoma. Ph.D. dissertation, Department of Biomedical Engineering, Duke University, 2006.

[44] Liang LL, Epstein DL, de Kater AW, Shahsafaei A, Erickson-Lamy KA (1992). Ethacrynic acid increases facility of outflow in the human eye in vitro.. Arch Ophthalmol.

